# Urdu translation and cross-cultural validation of Cumberland Ankle Instability Tool (CAIT)

**DOI:** 10.1186/s12891-022-05408-4

**Published:** 2022-05-12

**Authors:** Basma Khan, Mehwish Ikram, Syed Shakil ur Rehman, Zaib un Nisa

**Affiliations:** Faculty of Rehabilitation and Allied Health Sciences, Riphah International University, Islamabad, Lahore Campus, Pakistan

**Keywords:** CAIT, Ankle instability, Intraclass correlation, Validity, Reliability

## Abstract

**Background:**

The Cumberland Ankle Instability Tool (CAIT) is a self-assessment tool for people with chronic ankle instability (CAI). This tool had been translated and validated in many languages but there is no Urdu version of CAIT available.

**Objective:**

The aim was to translate the CAIT into the Urdu Language and determine its validity and reliability.

**Methods:**

A standardized step-wise forward and backward translation process was followed. Content, construct, convergent validity, internal consistency, and test–retest reliability were determined. A pilot study was done on 10 patients with CAI. The final version was investigated in 120 patients (mean age 26.6 ± 4.8 yrs) with CAI for validity and test–retest reliability in which 105 participants filled the questionnaire in the second week. Internal consistency was calculated by Cronbach’s alpha. Intraclass correlation (ICC_2,1_) was calculated to assess test–retest reliability between two weeks. Standard error of measurement (SEM) and smallest detectable change (SDC) were calculated. Convergent validity was determined by correlating Urdu CAIT with the Foot and Ankle Outcome Score (FAOS) using Spearman’s correlation co-efficient. Factor analysis describes the structure of underlying factors.

**Results:**

Content validity index was > 0.80 of each question. Internal consistency was acceptable (Cronbach’s alpha > 0.75). Convergent validity with FAOS total score showed a moderate negative correlation (r = -0.68) with U-CAIT and negatively correlated with subscales of FAOS. Test–retest reliability was excellent ICC_2,1_ > 0.80. Scree plot showed 3 factors > 1eigen value.

**Conclusion:**

The Urdu version of CAIT is a valid and reliable assessment tool for patients with chronic ankle instability. It has good content validity, construct validity and reliability.

**Supplementary Information:**

The online version contains supplementary material available at 10.1186/s12891-022-05408-4.

## Introduction

An Ankle sprain is a common injury and in layman’s terms is also known as a rolled or twisted ankle. It can occur to anyone, a common person working in his everyday routine or a sports athlete who plays tiring games involving speed, movement, and endurance [1]. An ankle sprain occurs when any one or more ankle ligaments are torn due to a sudden force or pull, placing the joint outside its normal functional range. If not treated on time, ankle sprains can lead to Chronic Ankle Instability (CAI) [2]. Ankle sprains are the most common injuries in sports and repetitive injuries lead to chronic ankle instability [3]. Often people ignore a sprained ankle which may increase the risk of developing chronic instability of the ankle [4].

A common way of determining CAI, and evaluating its effect on health status and quality of life, is to use a patient self-reported outcome measure. The self-reported ankle questionnaires include Foot and Ankle Ability Measure (FAAM) [5], Foot and Ankle Outcome Score (FAOS), [6] and the Cumberland Ankle Instability Tool (CAIT) [7]". The CAIT is an easy and understandable valid tool for the assessment of chronic ankle instability [7]. The CAIT can differentiate between the stable and unstable ankle. The original CAIT was developed in English and it showed a high content validity and test–retest reliability [7]. The CAIT has been translated into many languages e.g. Brazilian-Portuguese [8], Persian [9], Arabic [10], Korean [11], Japanese [12], Dutch [13], Greek [14], Chinese [15], and French [16]. Different versions of the CAIT were tested for internal consistency, test–retest reliability, ceiling and floor effects, and responsiveness [8–16].

Therefore due to the language hindrance and moderate pace of education, patients in Pakistan claim that it is difficult to comprehend English or another language survey. There are five territories of Pakistan and each territory has its language yet there is only one national language of Pakistan, Urdu. An official Urdu version of CAIT is not available. Therefore this study aimed to produce the Urdu version for Urdu-speaking countries and test its psychometric properties. An Urdu version of CAIT will have great significance for physiotherapists, educators, and researchers to assess and assist patients with chronic ankle instability.

## Methods

This cross-cultural validation study was conducted from January 2020 to January 2021 after the approval from Riphah International University and after the approval of the original author of CAIT. The study was started after the approval of the ethical committee from Riphah International University with a reference number REC/RCRS/20/1019.

### Tool Description (CAIT)

The CAIT consists of 9-items, with a total score of 30-points, for measuring the severity of functional ankle instability. A cut-off value of 27.5 was calculated by Hiller et al., (2006) to indicate ankle instability [7]. This means that all scores that are equal or less than 27 were considered unstable and the range above it was considered stable.

### Translation process

#### Guidelines

**Fig.1:**
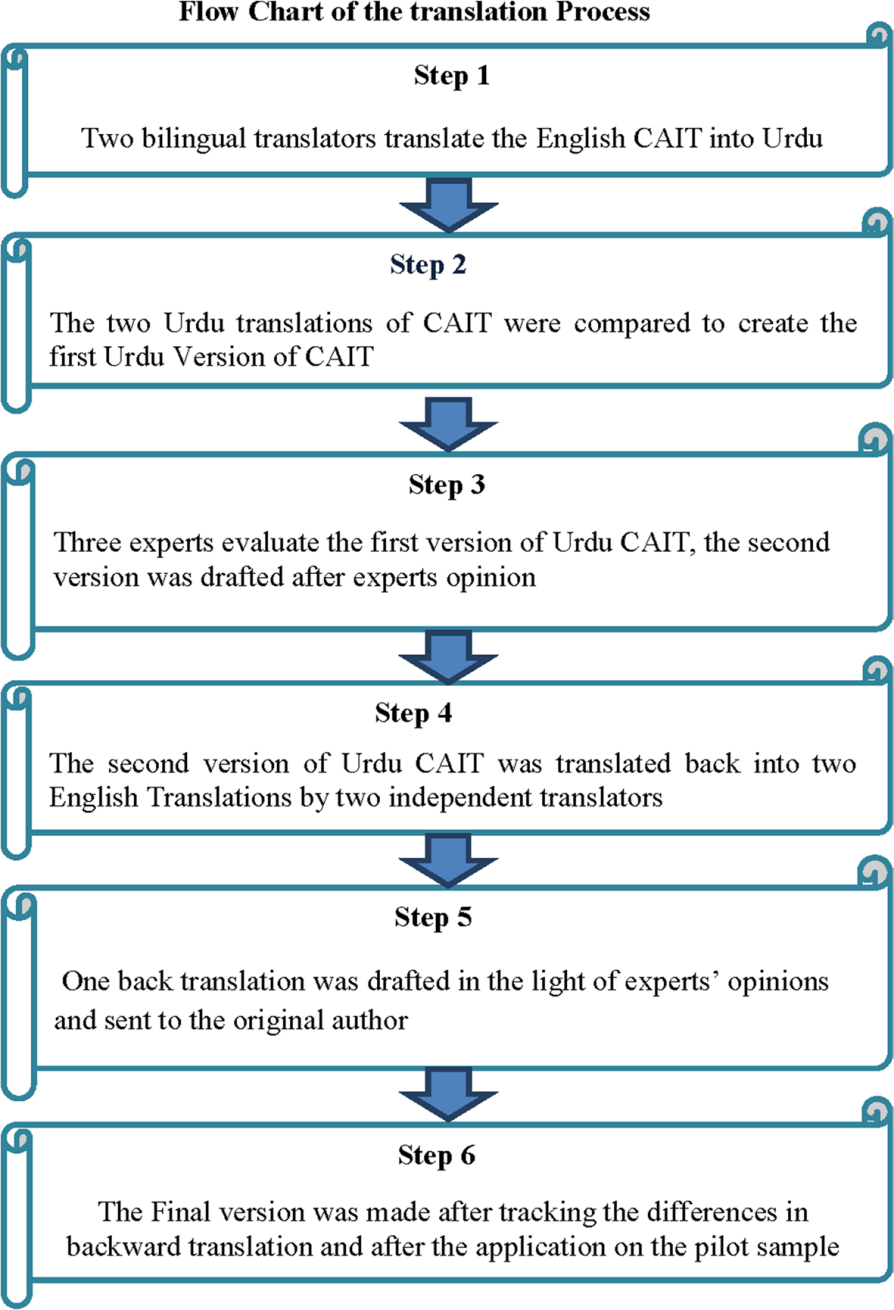
Flow chart of the translation process

A standardized translation method was used according to international guidelines (Beaton), followed in previous studies [17]. Validity and reliability were measured according to COSMIN guidelines [18]. All steps are shown in Fig. 1.

### Step 1: Forward Translation

CAIT was translated into Urdu by two bilingual translators (native) one was an informed (medical, a senior physical therapist) and the other was an uninformed (non-medical, expert in both languages English and Urdu) translator.

### Step 2: Experts Review Committee

Urdu was drafted after the consensus of the expert committee by modifying the required words to maintain the original concept of the questions (three experts who have experience of more than 10 years in rehabilitation were involved in the expert committee).

### Step 3: Back Translation

Urdu CAIT was translated back into English by two independent bilingual translators. Back translation was drafted by the consensus of the expert committee.

### Step 4: Experts Review Committee

The final version of the back translation was drafted after the experts’ review and sent to the original author for any further change. The percent agreement of three experts was noted in the whole process of translation shown in Table 1.

**Table 1:** Percent agreement of the experts in the translation process

Sr.No	Urdu-CAIT	Expert 1	Expert 2	Expert 3	Percent agreement
1	Question 1	3	3	3	100%
2	Question 2	3	3	3	100%
3	Question 3	3	3	3	100%
4	Question 4	3	3	3	100%
5	Question 5	3	3	3	100%
6	Question 6	3	3	3	100%
7	Question 7	3	3	3	100%
8	Question 8	3	3	3	100%
9	Question 9	3	3	3	100%

Abbreviations: *Sr. No *Serial Number, *CAIT *Cumberland Ankle Instability Tool

Table 1. Scoring of percent agreement of experts, 3: Complete agreement; 2: Partial agreement, and 1: No agreement.

### Step 5: Final Urdu Version

The final Urdu version was drafted after removing all the discrepancies. Content validity Index (CVI) and a pilot study were done.

- Content Validity Index of pre-final version
  The content validity of the pre-final Urdu version was determined by the clinical physical therapists (seven physical therapists were involved in this process). None of them were involved in the translation process. Content validity was determined; relevance, clarity, ambiguity, and simplicity, four subparts were further evaluated on the Likert scale, Waltz and Bausell method was adopted [19–21]**.** The content validity of instruments is also measured by several CVI. In this study, we have used 4 points ordinal content validity index developed by Waltz [21] to measure the content validity of Urdu CAIT. It has four categories: relevance, clarity, simplicity, and ambiguity and each of the categories of the CVI has been arranged on four points Likert scale.
- Pilot Study

A pilot study was done on 10 chronic ankle instability patients and healthy controls with no ankle instability. Discriminant validity was determined by comparing 10 patients with 10 healthy controls. The final version was drafted after application on the pilot data.

### Participants and data collection

Participants for the main study were recruited from patients having ankle problems analyzed as ankle instability and were treated by physiotherapists at different private athletic training institutes and hospitals of Multan (Multan Medical and Dental College, Nishter Hospital, Multan), Pakistan. A Convenience sampling technique was used.

Inclusion criteria for participants were: aged 20–50 years, able to understand and read the Urdu language, and suffering from ankle instability. For patients with complaints of turning of the ankle especially on uneven surfaces or in sports repeatedly, pain, swelling, tenderness, and persistent discomfort were included.

Exclusion criteria were: history of ankle surgeries, systemic and secondary ankle problems, central or neurological signs like paraesthesia or numbness, any red flags like night sweating, tumors or oncological issues, ankle arthrodesis, and ankle fractures.

Patients filled the Urdu-CAIT (U-CAIT) twice, in week 1 and week 2. FAOS was also filled in week 1 and its correlation was determined [6, 22].

### Data Analysis

SPSS version 25 (IBM, USA) was used for the data analysis.

In the pilot study, an independent t-test was applied to find out the discriminant validity between participants with and without chronic instability.

Internal consistency was determined by the Cronbach’s alpha [23], Intra class correlation (ICC_2,1_) for test–retest reliability [24].

For the main study, internal consistency was determined by using Cronbach’s alpha. The scores that are ranged from 0.50 to 0.69 are considered as poor, 0.70 to 0.79 acceptable, 0.80 to 0.89 good, and the value of Cronbach alpha that is > 0.90 is considered excellent [23].

Convergent validity was determined by the Spearman correlation with FAOS subscales (pain, stiffness, sports, and quality of life). Convergent validity was determined by correlating with other scales FAOS. Negligible correlation ranged from 0.00–0.10, weak correlation range from 0.10–0.39, moderate correlation range is 0.40–0.69, strong correlation range is between 0.70–0.89, and very strong correlation is between 0.90–1.00 [22].

Factor analysis was done to determine the Construct validity was determined by using principal component analysis (PCA) with Varimax rotation. The proportion of variances in the variables by the underlying factors can be determined by the Kaiser–Meyer–Olkin (KMO) measure of sampling adequacy and Bartlett's Test of Sphericity. [25–27].

Data summarization and data reduction are done in factor analysis and it is a powerful method for data reduction. Exploratory factor analysis describes the structure of underlying factors [25].

KMO is used for sampling adequacy to indicate the proportion of variance caused by the underlying factors and higher values that are close to 1 showed that the data is useful [27].

The factor loading for every item should exceed 0.5. The factor loading for every item should be 0.6 or higher according to Awang (2015). If any item has a factor loading less than 0.4 should be deleted from the measurement model [28].

Test–Retest reliability was measured with Intraclass correlation on week 1 and week 2 values. ICC_2,1_ values were calculated with 95% confidence intervals, values which were less than 0.5 indicated poor, 0.5–0.75 moderate, between 0.75 indicate good and > 0.90–1 excellent reliability [24].

## Results

### Translation and adaptation

#### Forward translation

No major issue was encountered during the translation of the questionnaire. In this study, the selected language Urdu is branched from Hindi, Persian, and Arabic and a big influence by English is observed on the regionally spoken Urdu language. For designing the final version of CAIT, all the factors were considered carefully.

### Back translation

The author of CAIT suggested some words e.g. choose the proper word which describes the difference between hop and bounce.

### Content validity index of Urdu CAIT

CVI of each question was ranged from 0.91–0.94. Scale content validity (S-CVI) was 0.92.

### Pilot study

The Pilot study was done on the 10 participants who have complaints of ankle instability shown in Table 2. In the pilot study, there was no change required in Urdu Version. Discriminant validity was determined by comparing them with healthy controls shown in Table 3. Independent t-test showed a significant difference in means of stable and unstable ankle participants. There was no change in the pre-final version so U-CAIT final version was drafted.

**Table 2:** Descriptive statistics of the pilot study on 10 patients with ankle instability

**Sr.No**	**Age** **(Years)**	**Gender**	**Marital Status**	**Ankle side**	**BMI**	**Urdu-CAIT**
1	35	Male	Married	Right	Overweight	17
2	30	Female	Married	Left	Obese	19
3	22	Male	Un-Married	Left	Overweight	16
4	23	Female	Un-Married	Right	Normal	21
5	29	Female	Married	Left	Normal	22
6	31	Male	Married	Right	Overweight	18
7	27	Male	Married	Right	Overweight	23
8	25	Male	Un-Married	Left	Obese	17
9	25	Female	Un-Married	Right	Normal	16
10	28	Female	Un-Married	Right	Overweight	21

Abbreviations: *Sr.No *Serial Number, *BMI *Body Mass Index

**Table 3:** Discriminative validity

Independent T-Test on Healthy Controls and Participants with chronic ankle instability					
	Gender	N	Mean	Std. Deviation	Std. Error Mean
Participants with CAI	M	7	20.00	2.38	0.89
	F	3	16.66	1.15	0.66
Healthy Controls	M	7	27.28	1.60	0.60
	F	3	27.33	0.57	0.33

Abbreviations: *CAI* Chronic Ankle Instability, *M* Male, *F* Female *Std* Standard

### Validity and reliability

Descriptive of the 120 participants with CAI are shown in Table 4.

**Table 4:** Descriptive statistics (demographics) of 120 patients with ankle instability

Descriptive statistics of 120 patients, *n* (%)	
Male	65 (55%)
Female	55 (45%)
Age, Mean ± SD	
Male	31.23 ± 1.19
Female	23.3 ± 3.28
BMI, Mean ± SD	
Male	27.2 ± 1.27
Female	28.5 ± 1.1
The affected side of the ankle, *n* (%)	
Right	79 (53%)
Left	59 (39%)
Both	12 (8%)

Abbreviations: *BMI* Body mass index, *SD* Standard deviation

Internal consistency of Urdu-CAIT at week 1 and week 2 was α ≥ 0.80 and correlation with FAOS was shown in Table 5. The mean values of each question ICC, SEM, SDC has shown in Table 6.

**Table 5:** Internal consistency, test–retest reliability, and validity analysis of the U-CAIT

U-CAIT Total (Mean ± SD)	
U-CAIT (Week 1)	15.5 ± 5.11
U-CAIT ( Week 2)	14.9 ± 5.16
Internal Consistency, Cronbach’s alpha	
Urdu-CAIT (Week 1)	0.79
Urdu- CAIT (Week 2)	0.77
Test–Retest Reliability: Intraclass correlation (ICC_2,1_)	
U- CAIT (Week 1 & 2)	0.82—0.95
Pearson Correlation of week 1 and week 2 readings	
CAIT (Week 1 & 2)	0.82—0.95
Convergent Validity (Spearman Correlations)	
FAOS Pain	-0.577
FAOS Symptoms/Stiffness	-0.597
FAOS Difficulty in daily activities	0.022
FAOS Sports and Recreation	-0.528
FAOS Quality of Life	-0. 782
FAOS Total	-0.678

Abbreviations: *U-CAIT* Urdu Cumberland Ankle Instability Tool, *ICC*_2,1_ Intraclass correlation, *FAOS* Foot and Ankle outcome Score

**Table 6:** Reliability analysis on 120 patients in week 1 and week 2

Urdu-CAIT	First Measurement Mean ± SD	2nd Measurement Mean ± SD	SEM	SDC	ICC_2,1_ (95% CI)	Item-total correlation U-CAIT	Cronbach’s Alpha
Question 1	2.8 ± 1.37	2.7 ± 1.34	0.087	0.81	0.95 (0.93–0.96)	0.90	0.949
Question 2	1.6 ± 1.23	1.5 ± 1.18	0.077	0.76	0.98 (0.96–0.98)	0.95	0.976
Question 3	1.2 ± 0.75	1.2 ± 0.64	0.042	0.56	0.93 (0.90–0.95)	0.87	0.929
Question 4	1.5 ± 0.75	1.5 ± 0.54	0.048	0.60	0.95 (0.93–0.96)	0.90	0.950
Question 5	1.3 ± 0.54	1.2 ± 0.51	0.034	0.51	0.90 (0.86- 0.93)	0.82	0.905
Question 6	1.0 ± 1.04	0.9 ± 0.98	0.065	0.67	0.94 (0.92–0.96)	0.89	0.944
Question 7	1.9 ± 1.04	1.8 ± 0.98	0.065	0.67	0.98 (0.96–0.98)	0.95	0.976
Question 8	2.1. ± 0.84	2.0 ± 0.83	0.056	0.61	0.92 (0.88–0.94)	0.84	0.918
Question 9	1.9 ± 0.86	2.0 ± 0.83	0.054	0.61	0.93 (0.90–0.95)	0.87	0.930
Total Score	15.5 ± 5.11	14.9 ± 5.16	0.331	1.59	0.94 (0.92–0.96)	0.89	0.948

Abbreviations; *CAIT* Cumberland ankle instability tool, *W1* Week 1, *W2* Week 2, *SD* Standard deviation, *SEM* Standard Error Mean, *SDC* Smallest Detectable Change, *ICC* Intraclass correlation]

**Fig. 2:**
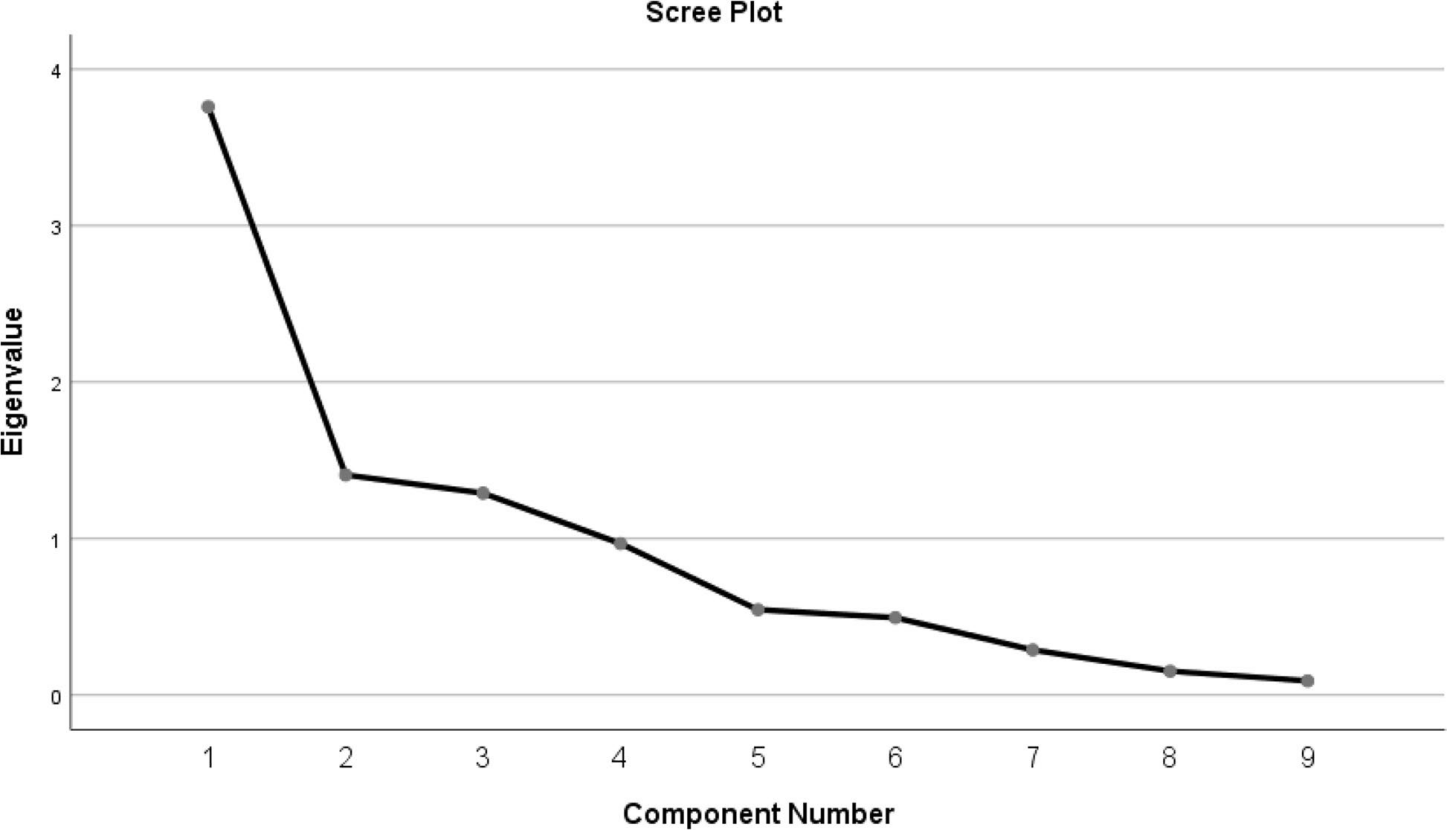
Scree plot

The factor analysis results showed that the KMO (measure of sampling adequacy) was 0.55 and Bartlett’s test was 569.7 (P < 0.01). The factor analysis and scree plot (Fig. 2) analysis showed that total variance was mainly described by three components. The cumulative variance for the 3 components (factors) was 71.77%. And Factor analysis readings are greater than 0.4 that is considered suitable shown in Table 7.

**Table 7:** Factor Loadings

Item		Factor
1	I have pain in my ankle	0.808
2	My ankle feels unstable	0.638
3	When I make sharp turns my ankle feels unstable	0.715
4	When I going down the stairs my ankles feels unstable	0.709
5	My ankle feels unstable when standing on one leg	0.581
6	My ankle feels Unstable	0.793
7	My ankle feels unstable	0.623
8	Typically when I start to roll over or twist on my ankle, I can stop it	0.776
9	After a typical incident of my ankle rolling over my ankle returns to normal	0.771

**Fig. 3:**
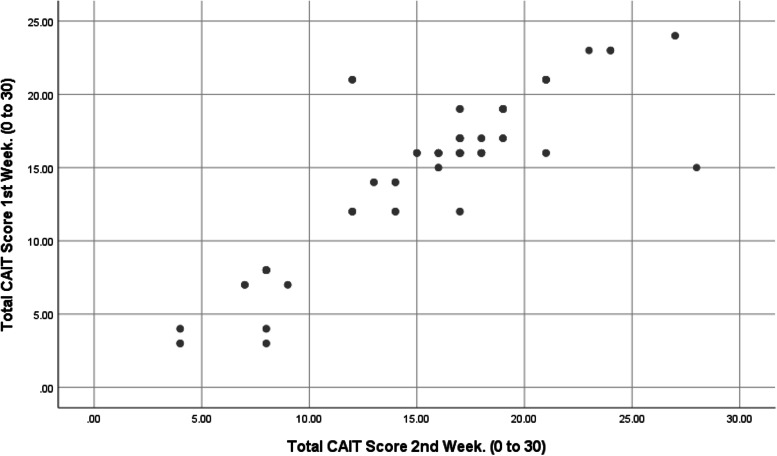
Scatter plot between week1 and week 2 U-CAIT

Intraclass correlation (ICC_2,1_) of each question was excellent and ranged from 0.90–0.96 with a 95% confidence interval. Scatter plot showed the linear relationship between week 1 and week 2 readings (Fig. 3).

## Discussion

The ankle sprain is the most common injury that occurred during physical exercise practice. When ankle sprains occurred due to weakness and pain some physical activities are restricted. With these permanent symptoms, the ankle instability becomes chronic and leads to chronic ankle instability [29]. CAIT is the most widely used patient-reported questionnaire for chronic ankle instability. Patient administrated tools are getting more importance in the assessment of physical activity, function, perception, and quality of life.

In the present study, CAIT was translated into Urdu language and found the version to have good content validity, moderate convergent validity, and internal consistency. The reliability was excellent. This new Urdu translated version will be used for the evaluation of functional ankle instability in Urdu-speaking patients.

It showed good content validity. Discriminant validity was determined to compare the results of patients with healthy controls. Urdu translated version of CAIT has good reliability and validity in patients with ankle instability. The test–retest reliability and internal consistency was calculated by ICC_2,1_ and Cronbach’s alpha (ICC_2,1_ = 0.93, α = 0.92). The intraclass correlation coefficient was calculated to measure the test–retest reliability; the value of ICC for total score was > 0.90 which indicates that CAIT has good test–retest reliability. Convergent validity was determined with FAOS and showed a moderate correlation with all subscales of FAOS but a low correlation with difficulty in daily activities. A moderate correlation was due to specific questions related to ankle stability in pain, symptoms, stiffness, and sports/recreational activities subscales. While in the case of difficulty in the daily activities (subscale) that contain more questions than other subscales, the relationship was low due to some activities in which sharp ankle movements were not required. As it is a patient-reported questionnaire and results were based on their perceptions.

Urdu-CAIT showed excellent internal consistency similar to previously translated versions, such as French [16], Dutch [13], and Spanish versions [26] but not as high as the Arabic language CAIT ( 0.92) [10]. Some versions of CAIT results showed good internal consistency of α > 0.85 [8, 11, 14, 15].

Urdu CAIT showed an excellent test–retest reliability > 0.90 and in previous studies found the same results as in Arabic Cumberland Ankle Instability Tool ( ICC = 0.75–0.98) [10] and > 90 as in Spanish[26], Dutch [13], Chinese-Taiwan version [15] and French version showed > 0.95 [16].

Convergent validity of the Urdu-CAIT was determined which showed a moderate (negative) correlation with FAOS. Dutch CAIT included the comparison with Foot and Ankle Outcome Score (FAOS), which showed a moderate correlation (0.36–0.43) [13].

In the evaluation of the construct validity, the exploratory factor analysis was done that showed and explained 71.8% variance, as original CAIT has shown no factor analysis but in the Spanish version, it was 66.4% [26].

Almost all the cross-cultural adaptation and validity studies were done for CAIT in the past as mentioned above followed the same method and procedure as we did for creating U-CAIT. The ICC values and other important parameters of U-CAIT such as validity, means, standard deviations, and errors were all relatable to the results of the other studies mentioned above. All the studies tested for the psychomotor properties of CAIT and provided a comparison with tools such as FAAM, FAOS, Visual Analogue Scale (VAS), or Numeric Pain Rating Scale (NPRS).

It will be beneficial for the clinicians in Pakistan and also in other Urdu-speaking countries.

Responsiveness was not measured because of the short duration between the two measurements. Data was collected from only patients with complaints of chronic ankle instability, proper screening methods were not applied. We recommended that it should be done on large scale to find out the cut-off value in the Asian population.

## Conclusion

Urdu version of Cumberland ankle Instability tool (U-CAIT) was drafted and used for the evaluation of chronic instability of ankles. U-CAIT showed good content validity and construct validity. Internal consistency and Test–retest reliability were found satisfactory. This tool is effective to be used to assess the ankle instability in the Urdu-speaking population.

### Implications to Physiotherapy

Cumberland Ankle Instability Tool is widely used nowadays for the assessment of chronic ankle instability. As it is a self-reported questionnaire and Urdu version of the Cumberland ankle instability tool will be beneficial both for patients and clinicians. It will also open research eras worldwide by comparing and sharing the results.

## Supplementary Information

**Additional file 1.** 

## Data Availability

The dataset used and analyzed during the current study is available from the corresponding author on reasonable request. It is not shown publicly due to privacy concerns.

## Abbreviations

CAIT: Cumberland Ankle Instability Tool
CAI: Chronic Ankle Instability
COSMIN: Consensus-based Standards for the selection of health Measurement Instruments
CVI: Content Validity Index
ICC: Intraclass Correlation
FAOS: Foot Ankle and Outcome Score
KMO: Kaiser-Meyer-Olkin
