# Supplementary material for: Urdu translation and cross-cultural validation of Cumberland Ankle Instability Tool (CAIT)
Source: BMC Musculoskelet Disord. 2022 May 12;23:443. doi: 10.1186/s12891-022-05408-4 (PMC9097047; doi:10.1186/s12891-022-05408-4)
Supplement: Supplementary file 1 — Additional file 1. [file 12891_2022_5408_MOESM1_ESM.pdf]

# CUMBERLAND ANKLE INSTABILITY TOOL

# کمبر لینڈ اینکل انسٹیبیلٹی ٹول

## غیر متوازن ٹخنے سے متعلق سوالنامہ

Urdu Version of the Cumberland Ankle Instability Tool-UCAIT

| | |
|---|---|
| **Please Tick The One Statement each Question that Best describes your ankle.** | **براہ کرم ہر ایک سوال کیلئے ایک عبارت کا انتخاب کریں جو آپ کے ٹخنے کی حالت کو صحیح طریقے سے واضح کرتی ہو۔** |

| سکور | دائیاں ٹخنہ | بائیاں ٹخنہ | | |
|---|---|---|---|---|
| | | | **میرے ٹخنے میں درد ہوتا ہے۔** | 1. |
| 5 | | | کبھی نہیں | الف |
| 4 | | | کھیل کے دوران | ب |
| 3 | | | ناہموار سطح پر بھاگتے ہوئے | ج |
| 2 | | | ہموار سطح پر بھاگتے ہوئے | د |
| 1 | | | ناہموار سطح پر چلتے ہوئے | ھ |
| 0 | | | ہموار سطح پر چلتے ہوئے | و |
| | | | **میرا ٹخنہ غیر متوازن محسوس ہوتا ہے جب۔** | 2. |
| 4 | | | کبھی نہیں | الف |
| 3 | | | کبھی کبھار کھیل کے دوران (ہمیشہ نہیں) | ب |
| 2 | | | اکثر کھیل کے دوران (ہمیشہ ہی) | ج |
| 1 | | | کبھی کبھار روزمرہ کے کام کاج کے دوران | د |
| 0 | | | اکثر روزمرہ کے کام کاج کے دوران | ھ |
| | | | **جب میں تیزی سے مڑتا / مڑتی ہوں تو میرا ٹخنہ غیر متوازن محسوس ہوتا ہے۔** | 3. |
| 3 | | | کبھی نہیں | الف |
| 2 | | | کبھی کبھار بھاگتے ہوئے | ب |

RIPHAH INTERNATIONAL UNIVERSITY

Translated and adapted by Basma Khan, Mehwish Ikram and Syed Shakil ur Rehman, Riphah International University, Islamabad (Lahore Campus).
Permitted by Claire Hiller

| | | | | |
|---|---|---|---|---|
| 1 | | | اکثراوقات بھاگتے ہوئے | ج |
| 0 | | | چلتے ہوئے | د |
| | | | **سیڑھیاں اترتے ہوئے میرا ٹخنہ غیر متوازن محسوس ہوتا ہے۔** | **.4** |
| 3 | | | کبھی نہیں | الف |
| 2 | | | اگر میں تیز چلوں | ب |
| 1 | | | کبھی کبھار | ج |
| 0 | | | ہمیشہ | د |
| | | | **میرا ٹخنہ غیر متوازن محسوس ہوتا ہے جب میں ایک ٹانگ پر کھڑا/کھڑی ہوتی ہوں جب** | **.5** |
| 2 | | | کبھی نہیں | الف |
| 1 | | | میں پنجوں کے بل کھڑا/کھڑی ہوں | ب |
| 0 | | | میں پورے پاؤں پر کھڑا/کھڑی ہوں۔ | ج |
| | | | **میرا ٹخنہ غیر متوازن محسوس ہوتا ہے۔** | **.6** |
| 3 | | | کبھی نہیں | الف |
| 2 | | | ایک طرف سے دوسری طرف اچھلتے ہوئے | ب |
| 1 | | | ایک خاص جگہ پر کودتے ہوئے | ج |
| 0 | | | جب میں چھلانگ لگاتا ہوں | د |
| | | | **میرا ٹخنہ غیر متوازن محسوس ہوتا ہے جب** | **.7** |
| 4 | | | کبھی نہیں | الف |
| 3 | | | میں ناہموار سطح پر بھاگتا ہوں۔ | ب |
| 2 | | | میں ناہموار سطح پر آہستہ بھاگتا ہوں۔ | ج |
| 1 | | | میں ناہموار سطح پر چلتا ہوں۔ | د |
| 0 | | | میں ہموار سطح پر چلتا ہو | ھ |
| | | | **عام طور پر جب میں ٹخنہ کے بل گھوموں یا مڑوں میں اسکو روک سکتا ہوں۔** | **.8** |
| 3 | | | فوراً | الف |

Translated and adapted by Basma Khan, Mehwish Ikram and Syed Shakil ur Rehman, Riphah International University, Islamabad (Lahore Campus).
Permitted by Claire Hiller

| | | | | |
|---|---|---|---|---|
| 2 | | | اکثر | ب |
| 1 | | | کبھی کبھار | ج |
| 0 | | | کبھی نہیں | د |
| 3 | | | میں کبھی ٹخنہ کے بل نہیں گھومتا | ھ |
| | | | **اگر کبھی میرا ٹخنہ مڑ جائے تو میرا ٹخنہ اصلی حالت میں واپس آجاتا ہے۔** | **.9** |
| 3 | | | تقریباً فوراً ہی | الف |
| 2 | | | ایک دن کے اندر اندر | ب |
| 1 | | | ایک سے دو دن میں | ج |
| 0 | | | دو دن سے زیادہ میں | د |
| 3 | | | میں کبھی ٹخنہ کے بل نہیں گھومتا۔ | ھ |

Translated and adapted by Basma Khan, Mehwish Ikram and Syed Shakil ur Rehman, Riphah International University, Islamabad (Lahore Campus).
Permitted by Claire Hiller
